# Integrated continuous biomanufacturing on pilot scale for acid‐sensitive monoclonal antibodies

**DOI:** 10.1002/bit.28120

**Published:** 2022-05-07

**Authors:** Hubert Schwarz, Joaquín Gomis‐Fons, Madelène Isaksson, Julia Scheffel, Niklas Andersson, Andreas Andersson, Andreas Castan, Anita Solbrand, Sophia Hober, Bernt Nilsson, Veronique Chotteau

**Affiliations:** ^1^ Dept. of Industrial Biotechnology KTH Royal Institute of Technology Stockholm Sweden; ^2^ AdBIOPRO, Competence Centre for Advanced BioProduction by Continuous Processing Sweden; ^3^ Dept. of Chemical Engineering Lund University, Lund Sweden; ^4^ Dept. of Protein Science KTH Royal Institute of Technology Stockholm Sweden; ^5^ BioProcess R&D, Cytiva Uppsala Sweden

**Keywords:** antibody aggregation, antibody manufacturing, Chinese Hamster Ovary cells, continuous chromatography, integrated continuous bioprocess, perfusion culture, Z_Ca_ ligand

## Abstract

In this study, we demonstrated the first, to our knowledge, integrated continuous bioprocess (ICB) designed for the production of acid‐sensitive monoclonal antibodies, prone to aggregate at low pH, on pilot scale. A high cell density perfusion culture, stably maintained at 100 × 10^6^ cells/ml, was integrated with the downstream process, consisting of a capture step with the recently developed Protein A ligand, Z_Ca_; a solvent/detergent‐based virus inactivation; and two ion‐exchange chromatography steps. The use of a mild pH in the downstream process makes this ICB suitable for the purification of acid‐sensitive monoclonal antibodies. Integration and automation of the downstream process were achieved using the Orbit software, and the same equipment and control system were used in initial small‐scale trials and the pilot‐scale downstream process. High recovery yields of around 90% and a productivity close to 1 g purified antibody/L/day were achieved, with a stable glycosylation pattern and efficient removal of impurities, such as host cell proteins and DNA. Finally, negligible levels of antibody aggregates were detected owing to the mild conditions used throughout the process. The present work paves the way for future industrial‐scale integrated continuous biomanufacturing of all types of antibodies, regardless of acid stability.

## Graphical abstract

A high cell density perfusion process, performed at pilot scale in 30 L bioreactor, was integrated with a continuous purification process including a Protein A capture using ZCa ligand with mild pH elution, a solvent/detergent‐based virus inactivation and two polishing steps, cation and anion exchange. This completely automated process generated purified antibody at very high yield with no detectable aggregation.

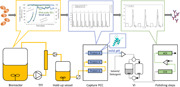

## 1 INTRODUCTION

Integrated continuous biomanufacturing of therapeutic proteins has gained a lot of interest over the past decade in the biopharmaceutical industry. In an integrated continuous bioprocess (ICB), a perfusion bioreactor with a mammalian cell line is coupled to a continuous downstream process (DSP). With increasing volumetric productivities from high cell density perfusion cultures and improved resin utilization in continuous chromatography, it offers potential economic benefits over conventional batch processing strategies (Pollock et al., 2017). Uncertainties in future market demands of biotherapeutic products have further led to questioning the lack of flexibility of large‐scale stainless‐steel plants (Walther et al., 2015). For example, the outbreak of a pandemic, such as recently the coronavirus disease (COVID‐19), can lead to capacity limitations of antibody production in fed‐batch processes (Coffman et al., 2021). The much faster implementation of an ICB in comparison to the construction of a new large‐scale stainless‐steel plant, allows for a rapid response in manufacturing to such public health threats. Scale‐out of several ICB units run in parallel as well as increase in total run time of the process, can rapidly be achieved if the demand for a biotherapeutic product changes (FDA, 2019), thus offering greater flexibility than traditional batch processes. This paradigm shift to integrated and continuous processing has been further expanded by including the design principles of using only single‐use equipment, closed processing and a “ballroom arrangement” in the new “biofacility of the future” (Klutz et al., 2015).

The earliest implementation of an ICB was demonstrated by Sanofi with a Chinese hamster ovary (CHO) cell perfusion culture linked to a four‐column periodic counter‐current chromatography (PCC) for continuous capture (Warikoo et al., 2012). This study paved the way for the development of end‐to‐end lab‐scale processes with integrated capture, virus inactivation (VI), and polishing steps, utilizing fully automated process control (Feidl et al., 2020; Godawat et al., 2015; Gomis‐Fons, Andersson, et al., 2020; Gomis‐Fons, Schwarz, et al., 2020; Steinebach et al., 2017). However, only a very limited number of studies have shown a successful demonstration of an end‐to‐end ICB on pilot scale. Arnold et al. described the integration of a 30 L perfusion culture with a multi‐column capture step, low‐pH VI and a filter train for product concentration, DNA removal and virus filtration (Arnold et al., 2019). In a pilot‐scale study by Coolbaugh et al. (2021) multi‐column chromatography was utilized in capture and polishing steps. Further downstream, continuous virus filtration, ultrafiltration and diafiltration (UF/DF) and product formulation were integrated into the process.

Tangential flow filtration (TFF) or alternating tangential flow filtration (ATF) perfusion bioreactors integrated to multi‐column capture chromatography have been widely applied as first units in an ICB. Process intensification through high cell density perfusion in TFF or ATF bioreactors with cell densities up to 130 × 10^6^ cells/ml has been demonstrated to boost the volumetric productivity of the upstream system (Chotteau, 2015; Clincke, Mölleryd, Samani, et al., 2013; Clincke, Mölleryd, Zhang, et al., 2013). The operation of a steady‐state culture with constant cell density can sustain the process with high productivity over a period of several weeks to months and simultaneously ensure a consistent quality of the product. Development of high cell density process from knowledge of an established fed‐batch process has shown a comparable glycoprotein quality profile between both operation modes (Särnlund et al., 2021).

The standard method used for the initial capture of antibodies from harvest is Protein A chromatography, a highly selective and efficient technique (Gagnon, 2012), but with detrimental effects on certain antibodies through the low‐pH elution step. At the typically used acidic elution pH of around 3.0–3.5, unwanted antibody aggregates can arise in the capture eluate, leading to compromised product yield and safety, with the required removal of these high‐molecular‐weight species further downstream in the manufacturing process (Liu et al., 2016; Paul et al., 2014; Shukla et al., 2007; Vázquez‐Rey & Lang, 2011). This especially affects up‐and‐coming therapeutics like IgG2 and IgG4 antibodies, which have a higher aggregation propensity (Hari et al., 2010; Ito & Tsumoto, 2013; Liu et al., 2016). Bispecific antibodies are also highly affected by the low pH since they suffer from aggregation of both active and inactive variants and often require several purification steps to remove these unwanted impurities (H. Li et al., 2020). This major drawback of Protein A chromatography can, however, be prevented by the use of a novel engineered Protein A ligand, called Z_Ca_, which is dependent on calcium ions for its binding to antibodies (Kanje et al., 2018). Depletion of calcium with a sodium chloride‐containing buffer results in the efficient release of the bound antibodies from the Protein A resin at close to neutral pH (6–7), differing slightly between the different subclasses of antibodies (Scheffel & Hober, 2021). These mild conditions prevented the formation of antibody aggregates during purification of an aggregation‐prone antibody. Customary washing with sodium chloride is still fully feasible by including a low concentration of calcium in the buffer, and high antibody recovery is obtained.

From the above‐mentioned ICB studies, a larger variation in designs has been proposed in the polishing steps, but most frequently one‐column and two‐column chromatography with cation exchange (CEX) and anion exchange (AEX) resins were implemented. Low‐pH VI in periodic batches or continuous plug‐flow reactors are in general the methods of choice for postcapture antibodies (Coffman et al., 2021). Changing a process from batch to continuous purification leads to similar product quality attributes, as revealed by a comparability study using similar parameters in both process modes (David et al., 2020). This will pave the way to adapt batch processing for clinical trial production to continuous large‐scale production.

The implementation of an ICB requires the full automation and control of the upstream and downstream systems. The environmental parameters in the upstream process are routinely monitored and controlled, while monitoring and control of nutrients, metabolites and proteins are approached by “Process Analytical Technology,” a vivid research field. The automation of the downstream process has a different challenge in terms of the multiplicity of the units relying on different equipment and control systems to be integrated. For that purpose, we have used the research software Orbit, previously developed for the control of integrated continuous downstream bioprocesses (Andersson et al., 2017; Gomis‐Fons et al., 2019; Gomis‐Fons, Schwarz, et al., 2020). Orbit saves user specifications, information about the downstream sequence, process events, signals, and data, thus allowing real‐time control and automation of all downstream units centralized in a single control system. Orbit also allows the implementation of complex flow paths in the chromatography systems and their synchronization, enabling simultaneous control of several systems and the integration of the downstream unit operations.

**Figure 1:**
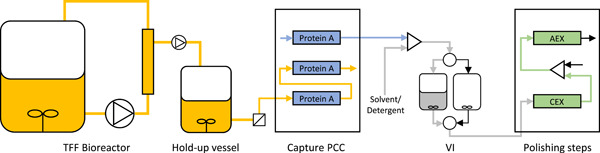
Process overview: on pilot scale, a 30 L TFF perfusion bioreactor was linked to a PCC system for the Protein A capture via an intermediate hold‐up vessel. Further downstream, a solvent/detergent‐based VI and two polishing steps with a CEX and an AEX resin are shown. AEX, anion exchange; CEX, cation exchange; PCC, periodic counter‐current chromatography; TFF, tangential flow filtration.

This article reports the results obtained from a pilot‐scale ICB with monoclonal antibodies (mAbs) produced in a 30 L high cell density perfusion bioreactor and simultaneous purification by a continuous downstream system. Here, the Z_Ca_ resin is applied in an ICB on pilot scale for the first time, as part of a three‐column PCC setup, with the subsequent VI step, based on a solvent/detergent method to avoid subjecting the antibodies to low pH throughout the process, and two polishing steps (Figure 1). High productivity and resin utilization are combined with mild purification of antibodies using this high‐capacity, calcium‐dependent resin. Moreover, the ability to remove process and product‐related impurities is here evaluated on pilot scale. Although the mAb used in this study was not particularly prone to aggregation at low pH, it provided a good model to assess the performance of the pilot‐scale process. The ICB proposed in this study was developed through collaborative effort between academia and industry with expertise in cell culture and purification process development, protein‐ligand design for chromatography and process automation. The feasibility for long‐term continuous process operation was validated at first in a small‐scale run with a 200 ml bioreactor. After final optimizations for enhancing process robustness, the ICB was scaled up by a volumetric factor of 150, using the same equipment for the downstream process, thus allowing a smooth and easy scale‐up. The pilot‐scale process was then successfully run in a test‐bed facility for biological production.

## 2 MATERIALS AND METHODS

### 2.1 Upstream system

#### 2.1.1 Cells and passaging

A CHO cell line with glutamine synthetase expression system producing Trastuzumab antibody was used in this study. This product is not characterized as pH‐sensitive mAb, and was used here as a model to demonstrate the operational feasibility of the unit operations. A cell line producing a more pH‐sensitive mAb was not available for this study. The cells used in the small‐scale experiments (KTH cell line) and in the pilot‐scale study (Cytiva cell line) were issued from research cell banks with different passage history, however with a common master cell bank as origin. The cells were routinely passaged or expanded in shake flasks (37°C, 5% CO_2_, 120 rpm).

#### 2.1.2 Culture media

Three cell culture media, all from Cytiva, were used for the cell expansion and/or the bioreactor cultures. HyClone ActiPro medium (HA), which contained 33 mM glucose, was used for cell expansion in shake flasks and bioreactor runs as mentioned in the text. HyClone ActiPro medium supplemented with 3% (w/w) Cell Boost 7a and 1% (w/w) Cell Boost 7b (HA + 7a/3 + 7b/1) contained 45 mM glucose. HyClone ActiPro medium supplemented with 6% (w/w) Cell Boost 7a and 1% (w/w) Cell Boost 7b (HA + 7a/6 + 7b/1) was also supplemented with glucose to obtain a final concentration of 73 mM glucose.

#### 2.1.3 Bioreactors and cell separation systems

The inoculations of the production bioreactors on small scale and pilot scale were carried out with cells from a perfusion seed bioreactor (N‐1 perfusion). On small scale, the seed and production bioreactors were identical systems, consisting of a stirred tank DASbox Mini Bioreactor system (Eppendorf) coupled with an alternating TFF system ATF2 (Repligen) with hollow fiber cartridge CFP‐4‐E‐3MA (Cytiva), operated with a recirculation flow of 0.5 L/min. A detailed description of this perfusion bioreactor system can be found elsewhere (Schwarz et al., 2020).

On pilot scale, the seed bioreactor was a wave‐induced bioreactor while the production bioreactor was a disposable stirred tank system. The N‐1 bioreactor was a 10 L WAVE perfusion Cellbag, mounted in a WAVE 25 rocking bioreactor system (Cytiva). The production bioreactor was a stirred tank Xcellerex XDR‐50 bioreactor operated at 30 L working volume, connected to a Xcellerex Automated Perfusion System (APS) for cell separation by TFF, where the cell culture was recirculating at 10 L/min through a RTPCFP‐4‐E‐9S hollow fiber with 0.84 m^2^ filter area and 0.45 µm pore size (all Cytiva).

#### 2.1.4 N‐1 bioreactor culture

Cells from shake flask cultures were inoculated at a viable cell density (VCD) of 1.5–2 × 10^6^ cells/ml in the respective N‐1 bioreactors and expanded over a course of 6–7 days to reach at least 60 × 10^6^ cells/ml. The cultures were controlled at setpoints of 37°C, pH 7.0 and a working volume of 0.2 or 5.0 L in the DASbox or the WAVE 25 bioreactor, respectively. The DO was maintained at 40% with a gas flow of 0.5 L/min with 21%–50% oxygen, in the WAVE 25 bioreactor. Once 50% oxygen was reached in the inflowing gas, the DO was controlled by variation of the rocking speed (22–28 rpm) at an angle of 6–7°. Perfusion at 0.25 vessel volumes per day (vvd) with HA medium was initiated when the cell density reached 5 × 10^6^ cells/ml. A target cell‐specific perfusion rate (CSPR) of 33 pl/(cell*day) was applied on the following days until the cell concentration target for transfer to the production bioreactor, ≥60 × 10^6^ cells/ml, was achieved.

#### 2.1.5 Production bioreactor culture

The cells from the N‐1 bioreactor were transferred to the production bioreactor with a target seed VCD of 10 × 10^6^ cells/ml. The setpoints for the temperature, pH and DO were 37°C, 7.0% and 40%. The pH was controlled with automatic additions of 0.5 M Na_2_CO_3_ or CO_2_ through a macro‐sparger in the XDR‐50 or via headspace in the DASbox bioreactor. In addition, in the XDR‐50 bioreactor, CO_2_ stripping was performed with up to 1.6 L/min airflow through a macro‐sparger to keep CO_2_ levels below 16 kPa. The DO was controlled at 40% by addition of oxygen up to 1 L/min through a micro‐sparger on pilot scale. A 3% antifoam C emulsion (Sigma‐Aldrich) was periodically added to prevent foam formation in the XDR‐50 bioreactor while no antifoam was used on small scale. Perfusion was initiated after inoculation of the bioreactors with a target CSPR of 25 pl/(cell*day) with HA + 7a/3 + 7b/1. At Day 4, medium was switched to HA + 7a/6 + 7b/1 and the perfusion rate was set to 1.5 vvd. A VCD of 100 × 10^6^ cells/ml was targeted from Day 5 after inoculation with a CSPR of 15 pl/(cell*day). The cell density was stabilized at this concentration by continuous cell bleeding.

### 2.2 Integration of upstream and downstream systems

The clarified harvest was collected in a stirred hold‐up vessel (HV), which was in turn connected to the downstream process. The HV acted as a buffer tank to account for harvest flow variations and to enable a continued operation of the perfusion bioreactor in case of downstream process interruption. On small scale the HV was a 1 L Duran bottle and on pilot scale, it was a 100 L sterile bag. To monitor the HV mass, a balance was used, which was a benchtop with serial interface on small scale and a floor balance with ethernet network interface on pilot scale. The chromatography systems were not sterile and, to ensure sterility upstream, two autoclaved 0.2 µm filters were connected in parallel in the line between the HV and the inlet to the chromatography system. These parallel filters were used one at a time and switched from one to the other one in case of filter clogging or malfunction. On small scale, these filters were CultureGard® HF Perfusion 0.2 µm filters (Repligen), and ULTA™ Pure HC 0.6/0.2 µm 5″ capsules (Cytiva) on pilot scale.

### 2.3 Downstream system

#### 2.3.1 Downstream unit operations and process conditions

The downstream process consisted of four steps (Figure 1): a Protein A capture step run with a 3‐column PCC process and with an in‐house developed calcium‐dependent Protein A resin (Z_Ca_); a solvent/detergent virus inactivation; a CEX step in bind/elute mode; and an AEX step in flow‐through mode. The two last steps are referred to as the polishing steps.

In the capture step, two columns were simultaneously loaded with clarified harvest, according to a 3‐column PCC operation. The breakthrough from the first column was loaded directly on the second one, hence, the product that did not bind to the first column could adsorb onto the second one, thus minimizing the product loss (Godawat et al., 2012). A third column was washed, eluted and regenerated while the other two were loaded. After loading a pre‐set volume of clarified harvest, the three columns switched positions and a new PCC cycle was initiated.

The process conditions (including flow rates and recovery times) and buffers were the same as described in previous work where the feasibility of the same downstream process on small scale was demonstrated (Scheffel et al., 2022). The elution of the capture step, the virus inactivation (VI) and the CEX step were performed at pH 5.5. Before the loading of the AEX column, the CEX eluate was diluted inline with a dilution ratio of 1:1 to adjust the pH and NaCl concentration to 6.2 and 120 mM, respectively. An alternative method for the sample conditioning is single pass diafiltration to reduce the buffer consumption and the volume of product (Rucker‐Pezzini et al., 2018), but it involves the use of an additional system. Thus, the dilution method was preferred due to its simplicity to implement.

For the virus inactivation, a solvent/detergent‐based method was used. Tri‐*n*‐butyl‐phosphate (Tnbp) was used as solvent at a concentration of 0.15 g/L and the detergent was Tween 20 at a concentration of 0.5 g/L. A stock solution with 1.5 g/L Tnbp and 5 g/L Tween 20 was prepared, and the capture pool was diluted with a ratio 1:9.

Cleaning‐in‐place (CIP) of the systems was performed every cycle with 1 M NaOH. In addition, the sample inlet valve, the sample pump, and the flow path that was in contact with the harvest were disinfected and cleaned every 2–3 h. This cleaning procedure consisted of the following steps: 1 M NaOH, 70% ethanol and equilibration buffer, with water flushes in between.

#### 2.3.2 Process scheduling

The operation of a PCC process interconnected with the VI and polishing steps requires process synchronization. In other words, a scheduling is needed to carry out the periodic downstream process smoothly without interruptions; and knowing the process times is essential for this task. The length of the recovery phases in the capture step was estimated to be around 40 min, and the length of the polishing steps was approximately 120 min. The two systems could therefore be synchronized so that three cycles of the capture steps were run simultaneously during one polishing cycle, as shown in the process Gantt diagram (Supporting Information: Figure S1). The virus inactivation bottles acted as hold‐up vessels to store the PCC elution pool. After three capture cycles, that is, pooling of three PCC elution peaks, the solvent/detergent stock solution was added to the corresponding VI bottle. According to our previous optimization study on the PCC process (Gomis‐Fons, Andersson, et al., 2020) a hold‐up vessel between the PCC step and the rest of the downstream process can provide a higher productivity than a process without this hold‐up vessel. As a matter of fact, in this latter case, there is a greater need of synchronization between the systems, which leads to unproductive waiting times. In addition, having two VI bottles facilitates the synchronization because one of the VI bottles can be filled with the product from the capture step, while the other VI bottle is emptied, as observed in the Gantt diagram. In this diagram, it can be seen that none of the chromatography systems have any waiting time, thus the utilization time of the systems is maximized, and the total processing time is minimized.

#### 2.3.3 Design of the process units

The column volumes needed for the three chromatography steps were calculated based on the protein load per volume of resin and the amount of product purified per cycle (Supporting Information: Table S1). The product amount was calculated based on the maximum expected harvest concentration and flow rate and the minimum loading length in the capture step, which corresponded to the length of all recovery phases in the capture step (40 min). As per the protein loads per volume of resin for the three columns, they were determined as described below. Regarding the VI step, two 50 ml bottles were used on small scale, and two 2 L bottles were used on pilot scale.

For the capture resin, a simplified method for the design of PCC processes was used to calculate the maximum possible protein load, based on the study on PCC design by Shi et al. (2021). In this study, it has been demonstrated that the operating binding capacity does not change significantly with the feed concentration if the breakthrough percentage of the first column during the interconnected load is kept constant and equal or lower than 50%. To estimate the protein load, a breakthrough curve experiment was performed with a harvest concentration of 1.45 mg/ml, a residence time of 2 min (close to the one obtained during the integrated runs), and a column volume of 1 ml (see breakthrough curve in Supporting Information: Figure S2). The protein load was obtained by calculating the area above the breakthrough curve and below the line defined by the feed concentration for a breakthrough percentage of 50%. This corresponds to the total amount of protein adsorbed on one column, which is equal to the total amount of product that can be loaded in a PCC cycle, assuming there is no product loss in the breakthrough. Regarding the protein load for the CEX and AEX steps, they were obtained from a case study from the manufacturer (Cytiva, 2015a). To account for capacity loss over time, 70% of the obtained protein loads were used for the column design, for all three resins.

The small columns were scaled‐up to pilot scale according to Kidal and Jensen's approach (Kidal & Jensen, 2006), which has been applied in previous publications (Gomis‐Fons et al., 2021; Hansen, 2017). This method is based on keeping the number of theoretical plates constant or higher by setting the residence time constant, but not necessarily the velocity, which provides flexibility to choose a column length that is more proper for pilot scale. Pressure constraints were taken into account to determine the column dimensions by using an empirical expression for the pressure drop over the columns depending on the velocity and the column length (Hansen, 2017). For the pilot‐scale run, HiScale™ 26/40 columns (diameter 26 mm, maximum length 40 cm) were used for the capture and the AEX steps, and HiScale 50/20 (diameter 50 mm, maximum length 20 cm) were used for the CEX step. For the small‐scale run, prepacked 1 ml HiTrap™ (diameter 7 mm, length 2.5 cm) columns were used for the three steps. All columns and resins, except for the Z_Ca_ resin, were provided by Cytiva.

The Z_Ca_ resin was produced and coupled as previously described (Scheffel et al., 2022). The resins used for AEX and CEX (Capto Adhere and Capto S ImpAct, respectively), were packed into the pilot‐scale columns according to the instructions of the manufacturer (Cytiva). The Z_Ca_ resin was packed according to the instructions for the MabSelect SuRe resin (since they share the same base matrix) but including 1 mM CaCl_2_ in the packing buffer to facilitate the proper conformation of the Z_Ca_ ligands. The column packing of each column was evaluated according to the instructions from the manufacturer on an ÄKTA pure 25 system, and the height equivalent to a theoretical plate (HETP) and the asymmetry factor was determined.

#### 2.3.4 Process setup

The downstream process was implemented in two chromatography systems, on both small and pilot scale: an ÄKTA™ pcc 75 for the capture step, and an ÄKTA™ pure 150 for the polishing steps (both Cytiva). Supporting Information: Figure S3 displays a detailed diagram of the downstream process with different flow paths that allowed the integration and automation of the process. The ÄKTA pcc was run with a 3‐column standard configuration with slight modifications. The sample pump was used to load the columns, pump A for washing, eluting, and regenerating the columns, and pump B and a versatile valve were used to dose the solvent/detergent stock solution into the two VI bottles. An additional versatile valve was used to collect the breakthrough from the second column during the interconnected load phase in a bottle. The outlet valve, which has several outlet ports, was used to send the product to either of the two virus inactivation bottles (thus using two ports for that), and to collect the eluate from the capture step in a bottle.

The ÄKTA pure was customized to enable the integration of the two polishing steps and the emptying of the VI bottles in a robust way. This setup was based on previous implementations of integrated column sequences in an ÄKTA pure system, and detailed descriptions to how two polishing steps can be integrated in one system can be found in a previously published article (Gomis‐Fons, Schwarz, et al., 2020). The main differences between the setup presented in the latter paper and the current work, is how the virus inactivation step was integrated in the ÄKTA pure system, and how the product was collected. The integration of the VI step was achieved by connecting tubing from the bottom of the VI bottles to an inlet valve, using one port per bottle. To empty a bottle, the corresponding position in the inlet valve was selected, and the CEX column was loaded with pump B. To ensure that the whole volume was loaded, the loading continued until the built‐in air sensor in the inlet valve detected air. After emptying a bottle, the tube would be filled with air until the inlet valve. To remove the air in the tube, pump A, connected to the air‐filled tube via a T‐cross as shown in Supporting Information: Figure S3, was used to reverse the flow and to fill the tube with buffer. Afterwards, the position of the inlet valve was changed back, and the loading continued for additional time to recover the product that was held up in the system. To enable product collection, two outlet valves were employed. One of them was used to collect the intermediate product from the VI and CEX steps, and the other one was used to collect the finished product from the AEX step. Product collection for analysis was performed daily after each of the downstream steps.

#### 2.3.5 Process control

The research software Orbit, developed at the Department of Chemical Engineering at Lund University, Sweden (Andersson et al., 2017), was used to control and automate the downstream process, based on user specifications about the real process, such as phase length, flow rate, and buffer valve positions. The instructions, phase information, process events, and signals are saved in Orbit, making it possible to run with real‐time control, where each action is triggered by an event. This allows for a completely automated process, where the user can monitor the process and modify the control parameters in case an adjustment is needed. Examples of the most relevant control parameters used in the downstream process are the volume of clarified harvest loaded on the capture step in each PCC cycle, the pool volumes or the flow rates used in each step. Orbit, written in Python, communicates with the chromatographic system ÄKTA via an application programming interface (also known as API) to send instructions at determined times and receive signals from the system. Orbit is not limited to one setup but can communicate with multiple systems and other additional equipment, such as the balance used to monitor the weight of the harvest tank. Examples of previous implementations of Orbit for real‐time control of multiple systems can be found elsewhere (Gomis‐Fons, Schwarz, et al., 2020; Löfgren et al., 2021). In a multi‐machine setup, each system is controlled by its own Orbit program, and they communicate with each other via handshaking. In other words, they use flags, which are Boolean variables that indicate whether a specific part of the process is ready or not. When the flags from both Orbit programs are active, they synchronize with each other, and the process continues.

#### 2.3.6 Stability study on the capture resin

The stability of the Z_Ca_ resin under continuous exposure to a CHO cell supernatant was studied over time, to obtain information about a suitable residence time in the column and the maximum number of purification cycles that can be efficiently run before the set of Z_Ca_ columns should be exchanged in the pilot‐scale run. Repeated purifications were carried out with the same process conditions as for the ICB but applied in small‐scale batch mode to one Z_Ca_ column, instead of in continuous mode using three capture columns. The different parameters were thus adjusted for the 1 ml HiTrap columns (Cytiva) that were used, previously coupled and packed by Cytiva after in‐house production. The loading time was doubled in each purification within the stability study to subject the resin to the supernatant for an equal amount of time as in the ICB, where each capture column acts as a second column of the loading zone followed by becoming the first column of the loading zone. A flow rate of 0.1 ml/min was used to minimize the volume of supernatant required, and the concentration of mAb was changed every 20 cycles. The CIP of the capture column was excluded from the procedure to limit the number of factors that could impact the resin lifetime. The purifications were continuously repeated for 114 cycles (>10 days).

### 2.4 Analytical methods

Samples from the bioreactors were taken every day to monitor the cell density and viability with a Norma XS cell counter (iPrasense) in the small‐scale cultures or a Vi‐CELL XR analyzer (Beckman Coulter) in the pilot‐scale cultures. Offline pH and pCO_2_ were measured with a ABL9 blood gas analyzer (Radiometer). The concentrations of glucose, lactate, and IgG were analyzed with a Cedex Bioanalyzer (Roche Diagnostics).

Samples were collected after the different unit operations including the bioreactor harvest, capture eluate, VI pool, CEX and AEX eluate. The IgG titer was regularly analyzed with a Cedex Bioanalyzer to estimate process yields at each step and the productivity of the ICB. N‐Glycosylation analysis of purified mAb (AEX eluate) was performed with a GlycoWorks RapiFluor‐MS N‐Glycan kit and an ACQUITY UPLC H‐Class system with a BEH Amide column (all Waters). The DNA content was measured in the harvest, capture, CEX and AEX eluate according to Scheffel et al. (2019) after each sample had been stored at +4°C for a couple of days. The HCP levels were analyzed in the same samples as previously described (Scheffel et al., 2022) where the details of the analytical method for the detection of Tnbp can also be found. In brief, the HCP levels were analyzed with a Gyrolab CHO‐HCP E3G kit on a Gyrolab system (Gyros Protein Technologies). The Tnbp content was analyzed in the product from the VI step, the CEX step (flow through and eluate) and the AEX step, using a reversed‐phase high‐performance liquid chromatography (RP‐HPLC) column with an RI detector as described in Scheffel et al. (2022).

To determine the aggregate content in collected samples, analytical size exclusion chromatography (SEC) was performed. Instrument setup was an Agilent 1260 HPLC with connected autosampler and diode array detector. A TSKgel G3000SWXL column from TOSOH Biosciences was used and the equilibration and separation buffer consisted of 50 mM sodium phosphate, 150 mM NaCl, pH 7. Samples of 20 µl were injected and the flowrate was set to 0.8 ml/min for 20 min. The Agilent raw data were imported into Python where the aggregate content could be determined by comparing monomer and aggregate peak areas at 280 nm.

Different analytical methods to detect a decrease of the capacity in the capture columns were implemented, as the capacity loss in these columns was considered to be a critical issue that could lead to significant product losses over time: (1) Real‐time inline measurement of the UV signal of the breakthrough from the second column during the interconnected load; (2) Daily offline analysis of the mAb concentration of the same stream; and (3) Daily offline analysis of the mAb concentration of the eluate and estimation of the yield in the capture step. With these measures, any decay in the capacity or unexpected product loss in the breakthrough streams that would lead to a decreased yield in the capture step could be detected.

## 3 RESULTS AND DISCUSSION

### 3.1 Upstream process

A small‐scale perfusion process with 0.2 L working volume integrated with a downstream process was performed to demonstrate the technical feasibility for long‐term operation of the novel ICB before pilot‐scale testing. The production process was operated in two stages; Stage 1 targeting to rapidly bring the cell density to 100 × 10^6^ cells/ml and Stage 2 aiming at maintenance of the system in a steady state at this cell density with high mAb productivity and low medium renewal. The N‐1 process was designed to rapidly increase the density of healthy cells and provide a high cell concentration into the production bioreactor to shorten the growth phase in the latter. The combination of selected perfusion rate and proportion of feed concentrates in the medium attempted to minimize the medium renewal while optimizing the cell growth during the cell expansion phases and the mAb productivity during the steady‐state production phase.

A common issue for the design of perfusion processes is the high glucose concentration of commercial media and feed concentrates, which can lead to the formation of high lactate concentration in the culture (Y. Zhang et al., 2015). We have previously presented a targeted feeding strategy denoted TAFE (L. Zhang et al., 2021), in which a target cell‐specific consumption rate (*q_glc*_target_) is selected by the operator, and from which the glucose concentration in the fed medium (including additives) is determined. Based on preliminary experiments it was observed that a strictly controlled sugar consumption with a *q_glc*_target_ of ≈ 1.1 pmol/(cell*day) enabled a low lactate formation while ensuring high mAb productivity and satisfying quality attributes.

For the seed bioreactor, the selection of a CSPR with 33 pl/(cell*day) using HA medium, which contains 33 mM glucose, allowed a *q_glc*_target_ of 1.1 pmol/(cell*day) according to the TAFE design. It was observed that HA medium could well support the cell expansion at a CSPR of 33 pl/(cell*day) up to 70 × 10^6^ cells/ml. However, the culture performance would be reduced at CSPR < 33 pl/(cell*day) unless reinforcement such as Cell Boost supplementation was carried out. In the production bioreactor, supplementation of the feed concentrates Cell Boost 7a and 7b was adopted since this medium reinforcement allowed to reduce the CSPR. HA + 7a/3 + 7b/1, which included 45 mM glucose, supported fast cell growth in the small‐scale trials, and was therefore selected for Phase 1. A CSPR of 25 pl/(cell*day) was compatible with *q_glc*_target_ of 1.1 pmol/(cell*day) considering a glucose concentration of 45 mM. During Phase 2 at steady‐state, priority was given to reduce the CSPR and growth rate, while maintaining the cell*‐*specific mAb productivity. Therefore, the CSPR was decreased to 15 pl/(cell*day) while a medium reinforcement was applied by increasing the Cell Boost 7a concentration. Using HA + 7a/6 + 7b/1, glucose was additionally supplemented to achieve a final concentration of 73 mM according to the TAFE design with a *q_glc*_target_ of 1.1 pmol/(cell*day). The present strategy for the selection of the media/feeds and glucose concentrations, including a TAFE approach, successfully generated a stable cell‐specific glucose consumption, and allowed stable operation of the perfusion process with minimized cell‐specific productivity of lactate (Supporting Information: Figure S4). A higher *q_glc* was obtained during the first 1–2 days of the processes, resulting from excessive glucose in the batch medium HA. The strategy for the medium selection applied in the present work is summarized in Table S2.

**Figure 2:**
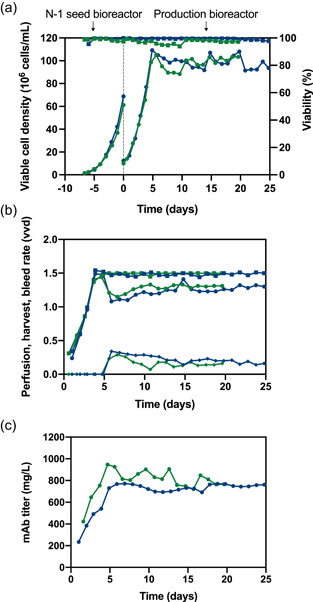
Perfusion culture data of the 0.2 L (blue) and 30 L pilot‐scale (green) run: (a) VCD (circles) and viability (squares) for N‐1 culture and production culture. (b) Harvest flow rate (circles), perfusion (feed) flow rate (squares) and bleed rate (diamonds). (c) mAb concentration in the harvest flow. mAb, monoclonal antibody; VCD, viable cell density.

The results of the perfusion cultures are shown in Figure 2. Successful scale‐up was achieved with a 150‐fold increase in the bioreactor working volume and perfusion flow despite differences in the cell retention systems, geometrical parameters of the bioreactors and aeration strategy. On both scales, high cell density cultures with 100 × 10^6^ cells/ml were maintained with high viability (>94%) (Figure 2a). The production bioreactors were inoculated from high cell density seed bioreactors operated in perfusion mode, which considerably shortened the cell expansion phase in the N‐stage bioreactors. As a result, the production process rapidly achieved the target cell density 4–5 days after inoculation. The production process was run for 20 and 25 days on pilot and small scale, respectively. When the cell density reached 100 × 10^6^ cells/ml, continuous cell bleeding was initiated with 0.1–0.3 vvd (representing 6.7%–20% of perfusion flow), while the perfusion rate was maintained at 1.5 vvd (Figure 2b). The harvest flow rate varied mostly between 1.2 and 1.4 vvd and the mAb titer in the harvest reached up to 1 g/L (Figure 2c). Thanks to proper adjustment of the nutritional depth in the perfusion medium, the low CSPR selected in the production runs (15 pl/(cell*day)) was sufficient to maintain a stable perfusion culture with high cell‐specific mAb productivity, low growth rate and high viability. Consequently, the product was more concentrated in reduced harvest volumes, which was beneficial for the process efficiency and for the subsequent capture chromatography.

### 3.2 Downstream process

The downstream process in the pilot‐scale run was started 2 days after starting the perfusion system, which allowed for building a buffer volume of the harvest in the HV. The clarified harvest was continuously purified for 17 consecutive days, from cultivation Day 2 until Day 19, aided by the control system Orbit to achieve process automation. During the initial days, the harvest mAb concentration was low, which resulted in a low concentration in the purified product. The harvest concentration increased until cultivation day 5, and after it had stabilized, the downstream process reached steady state. The downstream process in the small‐scale run was started 15 days after the start of the perfusion system, and run continuously for 9 days, from cultivation Day 15 until day 24.

**Figure 3:**
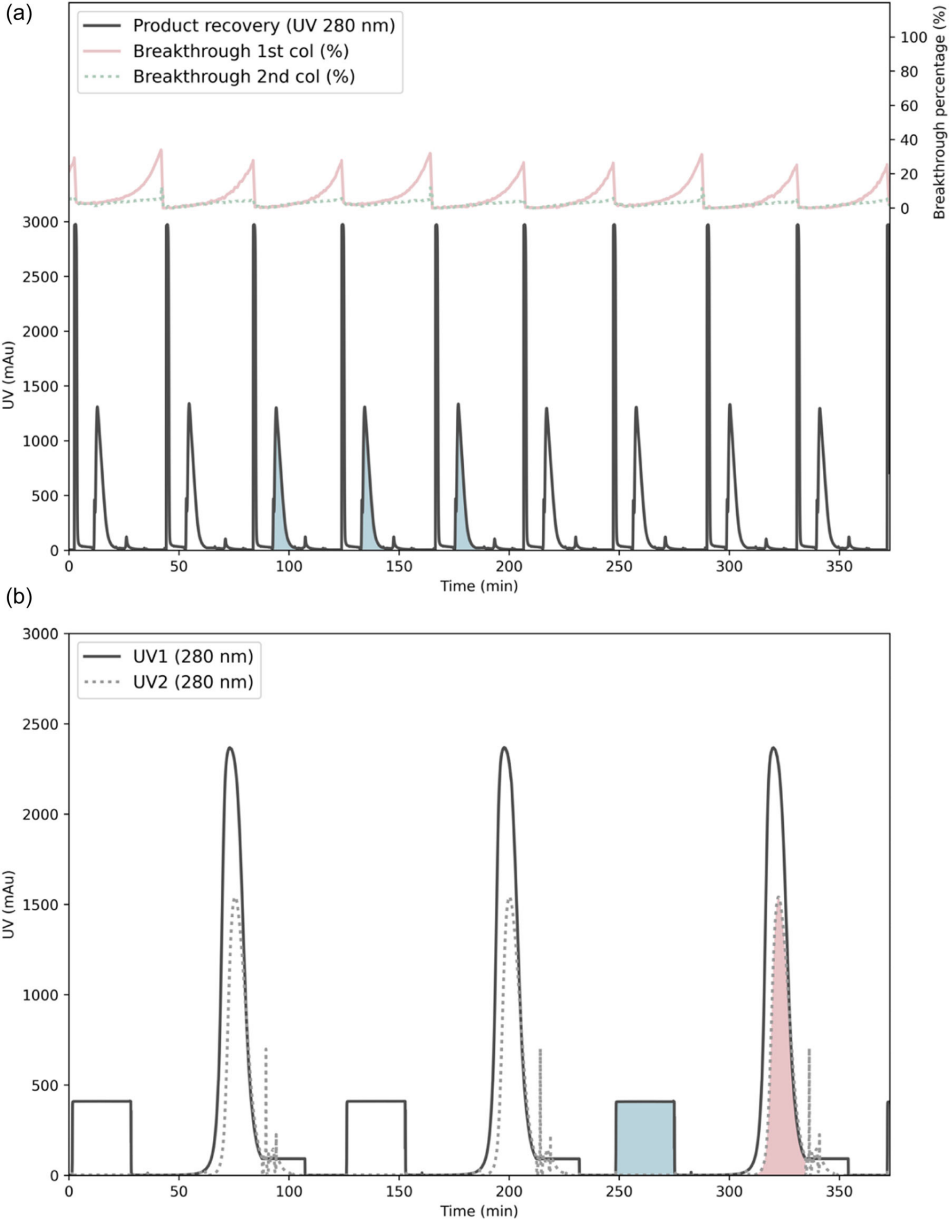
Steady‐state operation of the downstream process on pilot scale. (a) Capture step: UV signals from the three columns in the PCC operation. The breakthrough curves are expressed as percentages with respect to the feed concentration. The blue shaded peaks correspond to the product collected in the same VI bottle. (b) Polishing steps: UV signals from the two polishing steps. UV1 corresponds to the CEX column, and UV2 to the AEX column. The blue shaded area is the product loaded onto the CEX column, and the red shaded peak is the AEX eluate. AEX, anion exchange; CEX, cation exchange; PCC, periodic counter‐current chromatography; UV, ultraviolet.

In Figure 3, the operation of the downstream process on pilot scale during cultivation Day 15 is shown, illustrating several purification cycles over a couple of hours. In the capture step (Figure 3a), two large peaks can be seen during the product recovery step: the lower, broader peak corresponds to the elution of the product, and the higher peak corresponds to the wash phase, where the impurities are washed out from the column. The cycle time was around 42 min, and the recovery phases were, as mentioned, 40 min long. Hence, after product recovery in one of the columns, the loading of the two other columns continued for 2 min before changing the cycle. The breakthrough curves from the two columns that were loaded are also shown in the figure. The breakthrough curve from the first column reached levels of up to 30% during the interconnected load, with respect to the maximum level, which was the feed concentration. The PCC process was designed to be operated with a product breakthrough of 50% after the first column. However, the lower value in the breakthrough percentage obtained in the run was due to the fact that a 30% safety margin in the column capacity was considered, leading to a slight column oversizing. Regarding the breakthrough from the second column, the UV signal decreased only slightly during the loading, meaning that some product, although a small amount, was lost in that stream.

The chromatograms from the polishing steps are shown in Figure 3b. As previously explained, the polishing steps are run with product collected from three capture cycles, which is why the polishing cycles were longer. To enable process synchronization between the capture and polishing steps, the process time for the polishing steps should not exceed the time for three capture cycles. The polishing cycle started with the loading of the CEX column, which corresponds to the plateau in the UV1 signal in the figure. After the loading and a wash phase, the elution of the CEX column followed. The UV1 signal was measured after the CEX column and the UV2 signal after the AEX column. Since the AEX step was run in flow‐through mode, an increase in the UV absorbance could be seen on the UV2 sensor shortly after the UV1 sensor. Between the CEX and the AEX steps, there was a 1:1 inline dilution, meaning the loading flow rate in the AEX step was twice as high as the elution flow rate in the CEX step. As the figure shows the UV in relation to time, and not volume, the area under the peak from the UV2 signal is roughly half of that from the UV1 signal during this phase. The start and end of the pooling phases, from both columns, were automated using a pooling control strategy, which has been implemented in previous work (Löfgren et al., 2021). With this pooling control strategy, the pooling started at a predefined threshold UV value. Furthermore, a UV level cut‐off (i.e., the level at which the product pooling ends) was calculated based on the maximum peak height, which related to the product concentration. This way, the pooling strategy could adapt to varying incoming concentrations, which became especially important during the first days of cultivation, where the harvest concentration was still low. After the elution phase, the CIP, regeneration, and equilibration phases took place for both polishing columns in parallel. Pump A was used for the CEX column and the sample pump for the AEX column. During these phases, the UV2 monitor provided the signal from the AEX column while the UV1 monitor was not connected to an active flow path.

An important aspect in biomanufacturing is the product traceability, which is discussed in the guidelines on continuous biomanufacturing by the US Food and Drug Administration (FDA, 2019). In the case of continuous manufacturing, ensuring traceability means knowing the path that each specific portion of product followed through the downstream process. In a PCC operation, the product that is eluted is the one that was loaded in the previous cycle, but it also contains a fraction of the product that was loaded two cycles before, due to the interconnection of the columns in the loading phase. In Figure 3a, the product in the capture elution phase at around 100 min corresponds to product loaded during the two previous cycles (approximately the first 80 min). The product eluted from three capture cycles (at around 90, 135, and 175 min, corresponding to the blue shaded peaks in Figure 3a) was collected in one of the VI bottles. Then the solvent/detergent stock solution was dosed, and after 60 min of incubation, the product was purified in the polishing steps, corresponding to the polishing cycle starting at 250 min (blue shaded area in Figure 3b), and finally the product eluted at ca 330 min (red shaded peak in Figure 3b). Overall, the residence time of this portion of product in the downstream process was 330 min. In addition, the mean residence time in the HV (where the volume was kept at around 1 reactor volume and the flow rate was around 1.4 vvd) was ≈17 h. Since the HV was run as a continuous stirred tank, the residence time distribution could be estimated through simulation of an ideal stirred tank. Taking this into account, the processing of 99% of the product is up to five times the mean residence time, that is, ≈86 h, or 3.6 days. In total, the residence time of the product including the HV and the downstream process can be up to 91.2 h, almost 4 days. Hence, a deviation in the quality of the product entering the HV at a specific time could affect the finished product for up to 4 days. To deal with this situation, a valve before the HV was introduced in the flow path to divert the clarified harvest to an additional vessel in case a deviation was detected.

**Figure 4:**
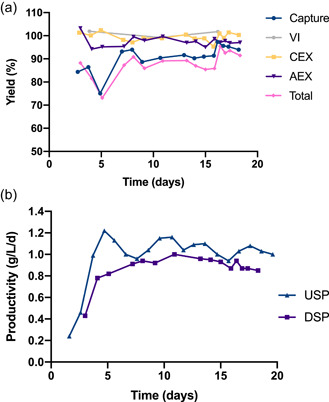
Recovery yield for the different downstream steps (a) and productivities (b) in the pilot‐scale run. AEX, anion exchange; CEX, cation exchange; DSP, downstream process; USP, upstream process.

Another important aspect in biomanufacturing is the recovery yield. In the presented downstream process, different yields were obtained on small and pilot scale. The total recovery yield on small scale was around 70%, with a maximum of 78% (Supporting Information: Figure S5). The process setup was not optimized for small scale, in particular not the method to empty the VI bottles. A substantial amount of product remained in the VI bottles after the loading was finished, which led to a decreased yield in that step. In addition, product loss in the loading of the capture columns occurred, which further reduced the total yield. On pilot scale, the process was operated to mitigate the mentioned issues and increase the total yield. The product loss during the emptying of the VI bottles was insignificant on pilot scale, and the aforementioned measures for estimating the product loss in the capture step allowed keeping a high yield throughout the pilot‐scale run, reaching levels of around 90%, with a maximum total yield of 95% (Figure 4a).

During the first days of the purification run on pilot scale, the harvest concentration was low; it increased from 0.2 to 0.9 g/L during the first 6 days. To compensate for the low initial concentration, the capture loading volume per cycle was set to 1450 ml, instead of the nominal value of 1188 ml. The higher initial loading volume, combined with a capture loading flow rate matching the initial low TFF perfusion flow, increased the capture cycle time from 40 to 60 min. On the first day, a total yield of almost 90% was achieved in the capture step. However, since the harvest concentration increased quickly, the yield decreased due to a higher amount of product loss in the breakthrough of the capture columns. To increase the yield, the loading volume was reduced to the nominal value (1188 ml), and once steady‐state operation was reached, a yield of 90% could once again be observed on cultivation Day 7. During Days 8 and 9, the capacity of the capture columns started to decrease slightly, and more product was detected in the breakthrough pool, exceeding the resin lifetime revealed by the resin stability study (Supporting Information: Figure S6). The columns had at this point been reused in approximately 300 cycles, which is a higher number of cycles than typically performed on Protein A resins, demonstrating the robustness of the Z_Ca_ resin (Rathore et al., 2015). The columns were exchanged on Day 9, and on the following days, the downstream process was running normally and with high yields. The yields of the individual steps were close to 100%, except for the capture step, where most of the product loss occurred. In particular, product was lost in the breakthrough stream, as can be seen in Figure 3, and this loss was estimated to 7%–10%. Without the use of PCC, the product loss in this step would have been considerably higher as the product flowing through the first capture column would have been lost instead of being adsorbed on the second capture column.

The operation of the pilot‐scale ICB resulted in a mean volumetric productivity of 0.92 g/(L*day) during the steady‐state phase of the perfusion, with a maximum of 1 g/(L*day), while the difference between the downstream productivity and the upstream productivity was small due to the high yields (Figure 4b). Considering the working volume of the bioreactor, which was 30 L, the ICB produced on average 28 g of mAb per day. The total production of purified mAb during 20 days of operation was around 470 g. Given the high productivity obtained and the comparatively long lifetime of the Z_Ca_ resin, this process is economically competitive with other ICBs based on conventional Protein A resins.

### 3.3 Product quality and purity

**Figure 5:**
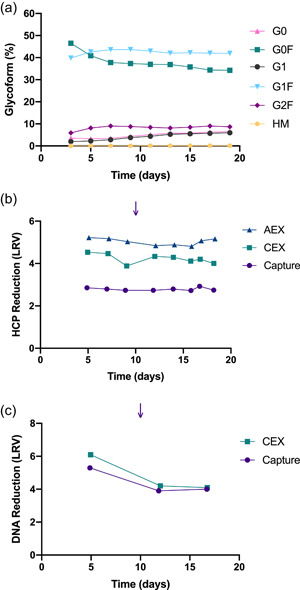
Quality and purity of the final product obtained in the pilot‐scale ICB throughout the entire run. The arrows mark the time point where the capture columns were switched. (a) The mAb glycosylation pattern indicating the percentage of the different glycoforms. (b) HCP log reduction values (LRV) demonstrating the removal of HCP after each purification step of the downstream process as compared to the initial harvest. (c) LRVs for DNA after capture and CEX at three‐time points throughout the process. LRVs for AEX is not included since the DNA levels were below the limit of detection. AEX, anion exchange; CEX, cation exchange; HCP, host cell proteins; ICB, integrated continuous bioprocess; mAb, monoclonal antibodies.

The steady‐state operation of the ICB ensured a stable product quality profile of the purified mAb. With regard to the N‐glycosylation profile of mAb, a shift to a higher percentage of galactosylation (G1F, G2F) occurred during the first days of the process (Figure 5a). From Day 7, the glycosylation pattern remained fairly stable due to the steady‐state operation of the bioreactor. It should be noted that high cell density inoculation of the bioreactor from a N‐1 perfusion culture reduced the transition period to a steady‐state from 10 to 7 days, compared to our previous work (Gomis‐Fons, Schwarz, et al., 2020). This shift in product quality could potentially be further accelerated by discarding the harvest from the first days of the culture, considering that the accumulated product has a residence time of up to 4 days, as discussed above. In addition, the setpoint of the harvest volume in the HV could be reduced to decrease the residence time of the product in this step. For example, a reduction of the HV volume to 0.1 reactor volumes would reduce the residence time from 91.2 to 14.1 h.

The removal of host cell proteins (HCPs) and DNA is a vital part of the downstream process, as these impurities make up a large part of all contaminants in an antibody production process, compromising the safety and quality of the final product (Y. Li, 2017). The CHO‐HCP content was quantified in the harvest as well as in the output from each purification step at several time points. Efficient HCP removal was seen for both the capture step and the two polishing steps, with a ca 5‐log reduction in the final product as compared to the harvest (Figure 5b). After the capture step using the Z_Ca_ resin, the impurity levels were reduced from >10^5^ ppm to on average 300 ppm, meaning approximately a 3‐log reduction, which is expected from commercial Protein A resins (Cytiva, 2015b; Scheffel et al., 2019). A further decrease is demonstrated after CEX, with HCP levels of on average 13 ppm and finally 2 ppm after AEX, which is low in comparison to other mAb processes (Cytiva, 2015b). Moreover, the DNA clearance was highly effective with high DNA log reduction values (LRVs) for the first set of capture columns on pilot scale, also leading to a higher LRV after CEX (Figure 5c) (Butler et al., 2009). There was a slight decrease in LRV at Day 10, but despite this, the DNA levels were below the detection limit after AEX throughout the entire run.

Regarding the virus inactivation, no experiments were performed to analyze the presence of virus in the product, but the implementation and efficacy of the solvent/detergent VI method has already been described in several publications (Martins et al., 2019; Orozco et al., 2017). Dichtelmüller et al. performed a comprehensive study of the virus inactivation process conditions, which was the base for the conditions selected in the present work (Dichtelmüller et al., 2009). According to this study, a concentration of 3 g/L Tnbp and 10 g/L detergent, and an incubation time of 60 min are the most common production conditions and enough to reach >5‐log inactivation, where a 4‐log inactivation is considered acceptable. The feasibility of this downstream process on small scale with the above‐described solvent/detergent concentrations has been demonstrated (Scheffel et al., 2022). In the pilot‐scale run, however, both concentrations were decreased to minimize the consumption and disposal of Tnbp, which is a hazardous and environmentally unfriendly compound. According to Dichtelmüller's study, a 4‐log inactivation can be achieved even at the lower concentrations used in the pilot‐scale run (Dichtelmüller et al., 2009).

The solvent used in the VI step, Tnbp, was not detected in the final product after AEX chromatography. The results show that Tnbp was successfully removed during the CEX chromatography step with the flowthrough, while the product in the CEX pool did not contain any detectable Tnbp residues (Supporting Information: Figure S7). The detergent used in the VI step, Tween 20, could not be detected in the purified product. However, it is a biocompatible agent and it is even commonly used in the formulation of monoclonal antibodies (Y. Li et al., 2014).

mAb aggregate levels after different steps in the downstream process have been monitored by SEC. The mild conditions of the capture PCC step and VI by solvent/detergent, with a pH of 5.5 instead of 3.2–3.5 which is conventionally used in a traditional mAb process (Arnold et al., 2019; Baur et al., 2016), resulted in insignificant levels of aggregates and fragments during the whole process after each unit operation with a maximum percentage of 0.3% aggregates and no fragments after CEX, and a concentration lower than the detection limit after AEX (Supporting Information: Figure S8). For comparison, typical values of aggregate levels in the purified product obtained in integrated continuous mAb processes range between 0.3% and 2.5% (Cytiva, 2015a; Godawat et al., 2015; Steinebach et al., 2017; Warikoo et al., 2012). In previous work, the Z_Ca_ resin was compared to commercial MabSelect SuRe for purification of IgG4, resulting in no aggregates for the Z_Ca_ resin and a substantial amount of aggregates for MabSelect SuRe with acidic elution conditions (Scheffel & Hober, 2021).

## 4 CONCLUSIONS

In this study, we presented a pilot‐scale demonstration of a fully continuous ICB for the production of a therapeutic mAb, which was operated for 17 days. The total run time could have been extended since no sign of deterioration of the process performance was observed until the end of the run. The steady‐state operation of the perfusion bioreactor ensured a continuous flow of harvest with consistent product quality and relatively stable mAb titer. The culture process was designed to reduce the time devoted to the cell expansion, ensure high mAb productivity, low medium renewal and high cell viability during the steady‐state phase at very high cell density of 100 × 10^6^ cells/ml. Thanks to the present rational approach for the selection of the medium, feed concentrates, glucose concentration and renewal rate, a healthy culture with a low level of the toxic by‐product lactate was obtained at low CSPR. Furthermore, the whole upstream process from the perfusion seed culture to the production bioreactor was performed with single‐use equipment.

A novel downstream process was integrated for the continuous purification of mAb under mild conditions using the novel Protein A ligand, Z_Ca_, which enables elution with a sodium chloride buffer at close to neutral pH. The feasibility of Z_Ca_ for antibody capture, connected to a subsequent VI step using a solvent/detergent‐based method instead of acid, was successfully demonstrated for the first time in a continuous downstream process on pilot scale. The Z_Ca_‐based capture step was able to continuously process the high titers from the upstream perfusion reactor for 300 purification cycles before switching to new capture columns, initiated by both real‐time inline and offline monitoring of potential capacity losses. The manufactured mAb contained very low impurity levels of aggregates, HCP and DNA, while the overall yield of the process was among the highest reported for mAb purification (Arnold et al., 2019; Godawat et al., 2015; Kamga et al., 2018; Steinebach et al., 2017). Aggregate formation was negligible and consequently contributed to a minimization of product loss. As noted, the model antibody used in this study is not very susceptible to aggregation. This process could, however, be applied to any antibody, which could greatly increase process yields since low‐pH elution and incubation can cause aggregation in a wide variety of products. The steady‐state operation of the ICB was guaranteed by the fully autonomous implementation of a centralized control system (Orbit), responsible for the synchronized operation and control of multiple integrated unit operations.

As the demand of biotherapeutics is changing, more cost‐effective and flexible production approaches are needed. We believe that ICBs utilized in manufacturing will become an attractive alternative to existing stainless‐steel fed‐batch facilities. This pilot‐scale study will take a step forward in the realization of production‐scale ICBs for commercial manufacturing of biologics, including acid‐sensitive mAb therapeutics.

## CONFLICT OF INTEREST

The authors declare the following financial interests/personal relationships which may be considered as potential competing interests: SH has filed a patent application regarding the novel Z_Ca_ domain.

## Supporting information

Supporting information.

## Data Availability

Data available upon reasonable request from the authors
