# Supplementary material for: Integrated continuous biomanufacturing on pilot scale for acid‐sensitive monoclonal antibodies
Source: Biotechnol Bioeng. 2022 May 7;119(8):2152–66. doi: 10.1002/bit.28120 (PMC9541590; doi:10.1002/bit.28120)
Supplement: Supplementary file 1 — Supporting information. [file BIT-119-2152-s001.pdf]

Supplementary Information for

Integrated continuous biomanufacturing on pilot scale for acid-sensitive monoclonal antibodies

Hubert Schwarz[a,e,*], Joaquín Gomis Fons[b,e,*], Madelène Isaksson[b,e,*], Julia Scheffel[c,e,*], Niklas Andersson[b], Andreas Andersson[d,e], Andreas Castan[d,e], Anita Solbrand[d,e], Sophia Hober[c,e], Bernt Nilsson[b,e,#], Veronique Chotteau[a,e,#]

[a] Dept. of Industrial Biotechnology, KTH Royal Institute of Technology, Stockholm, Sweden

[b] Dept. of Chemical Engineering, Lund University, Lund, Sweden

[c] Dept. of Protein Science, KTH Royal Institute of Technology, Stockholm, Sweden

[d] Cytiva, Uppsala, Sweden

[e] AdBIOPRO, Competence Centre for Advanced BioProduction by Continuous Processing, Sweden

*Co-first authors with equal contribution

[#] Co-corresponding authors

Table of Contents

Figure S1… ……………………………………………………………………………………………………..II

Figure S2…………………………………… …………………………………………………………………..II

Figure S3……………………………… ……………………………………………………………………….III

Figure S4…………………………… …………………………………………………………………………..IV

Figure S5…………………………… …………………………………………………………………………..IV

Figure S6………………………… ……………………………………………………………………………….V

Figure S7………………………… ……………………………………………………………………………….V

Figure S8…………………………… …………………………………………………………………………..VI

Table S1………………………… ……………………………………………………………………………….VII

Table S2………………………… ……………………………………………………………………………….VII

Supplementary Figures

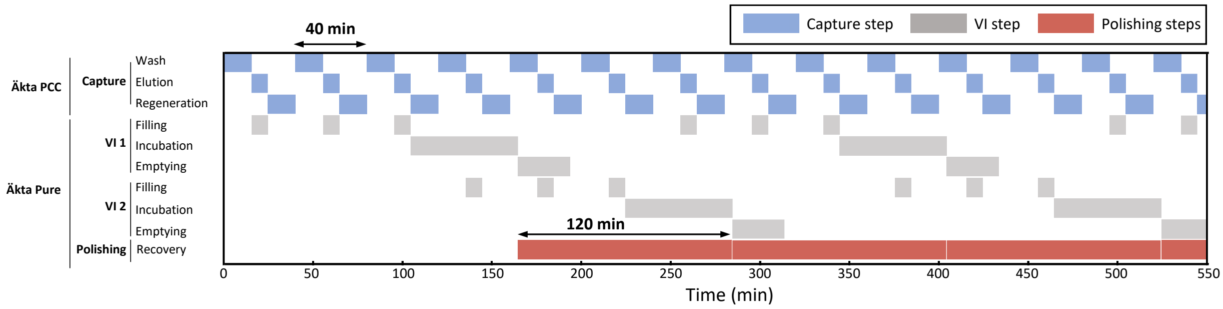

Figure S1. Gantt diagram of the downstream process.

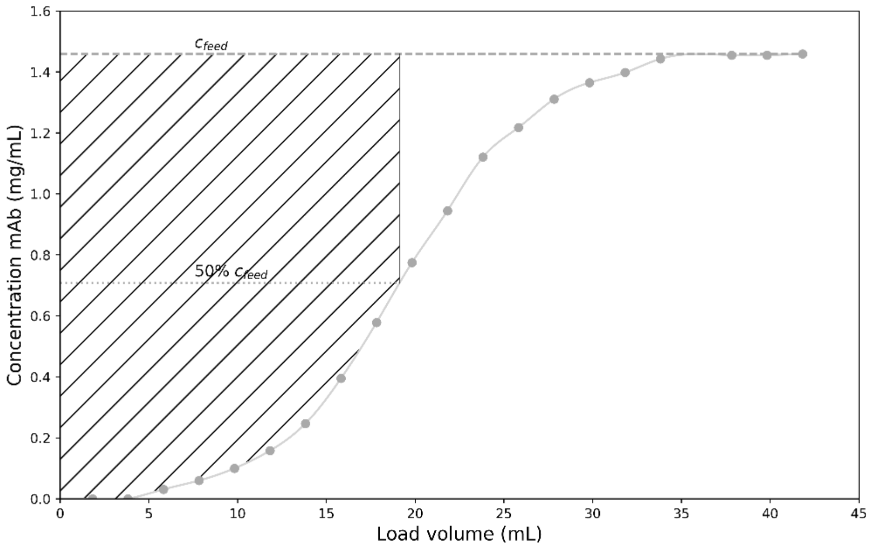

Figure S2. Breakthrough curve for the Z_{Ca} resin. The experiment was run at a harvest concentration of 1.45 mg/mL and a residence time of 2 min. A 1 mL HiTrap column (0.7 x 2.5 cm) was used. The marked area corresponds to the calculated protein load (in mg) at 50% breakthrough used in the column design.

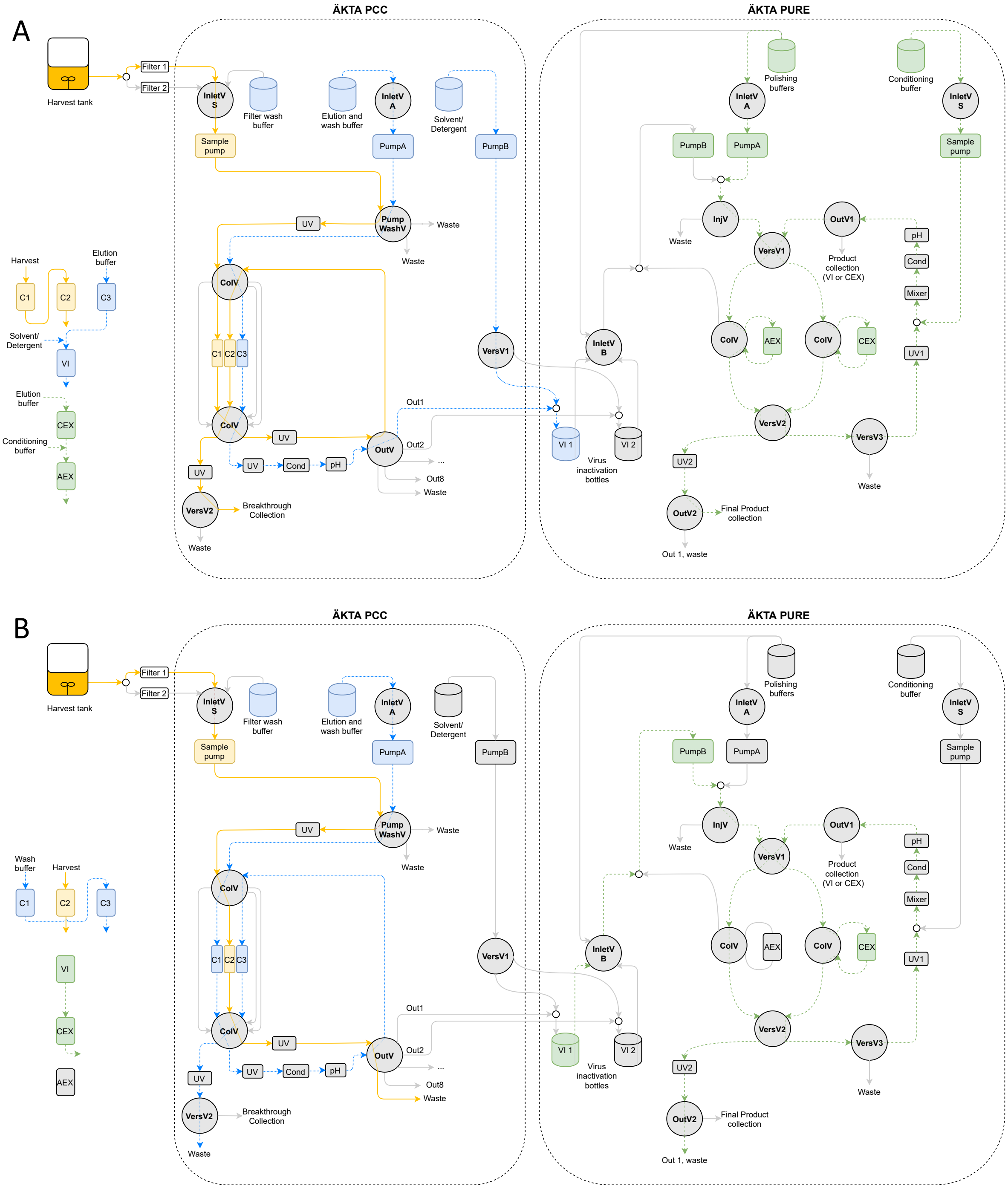

Figure S3. Detailed diagram of the downstream process setup. A) To the left, the flow path in the ÄKTA pcc system when two capture columns are loaded simultaneously (orange continuous line) and a third column is eluted (blue dotted line). To the right, the flow path in the ÄKTA pure system when the CEX column is eluted and the AEX is loaded with an inline dilution using the sample pump (green dashed line). B) To the left, the flow path in the ÄKTA pcc system when a capture column is loaded (red continuous line) and two capture columns are interconnected during the wash phase (blue dotted line). To the right, the flow path in the ÄKTA pure system when the CEX column is loaded from the VI bottle (green dashed line). Legend: OutV, outlet valve; VersV, versatile valve; InjV, injection valve; InletV, inlet valve; ColV, column valve; PumpWashV, pump wash valve; C, capture column; VI; virus inactivation bottle; CEX, cation exchange chromatography column; AEX, anion exchange chromatography column.

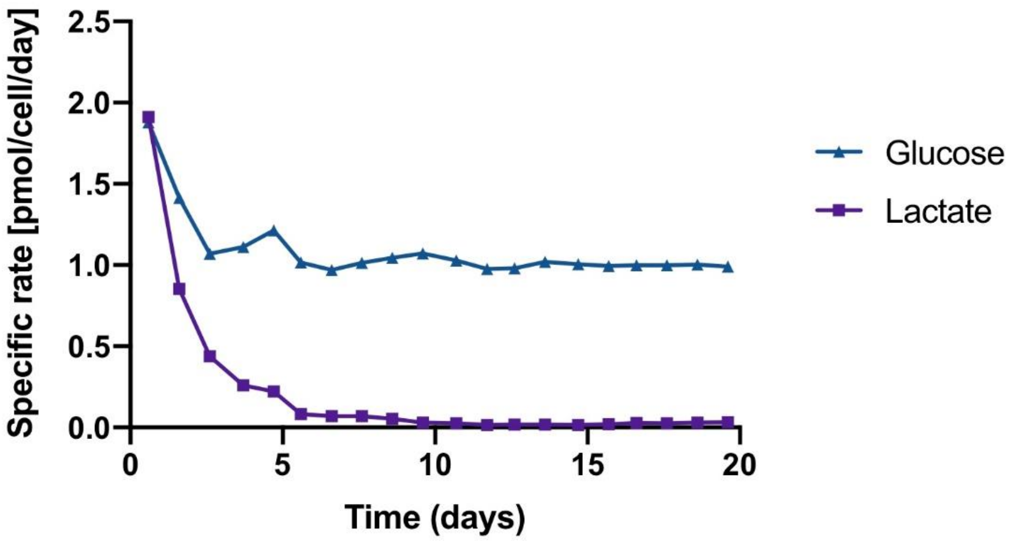

Figure S4. Cell specific glucose consumption and lactate production rate in the pilot-scale run. A constant glucose consumption rate of 1 pmol/(cell*day) was targeted.

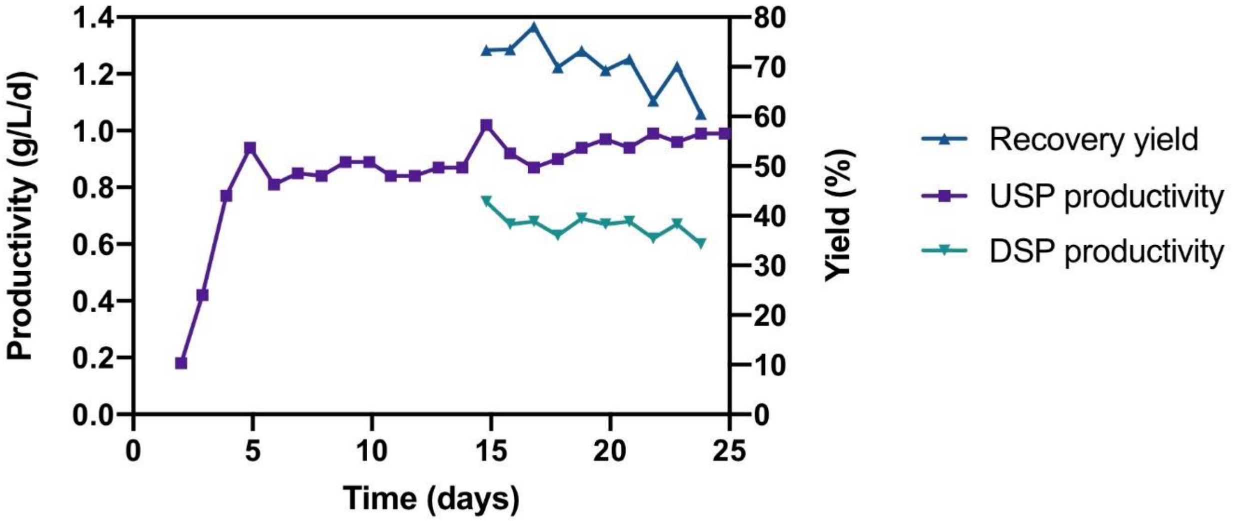

Figure S5. Recovery yield and productivity in the small-scale run. Productivities are expressed as grams per day and L bioreactor.

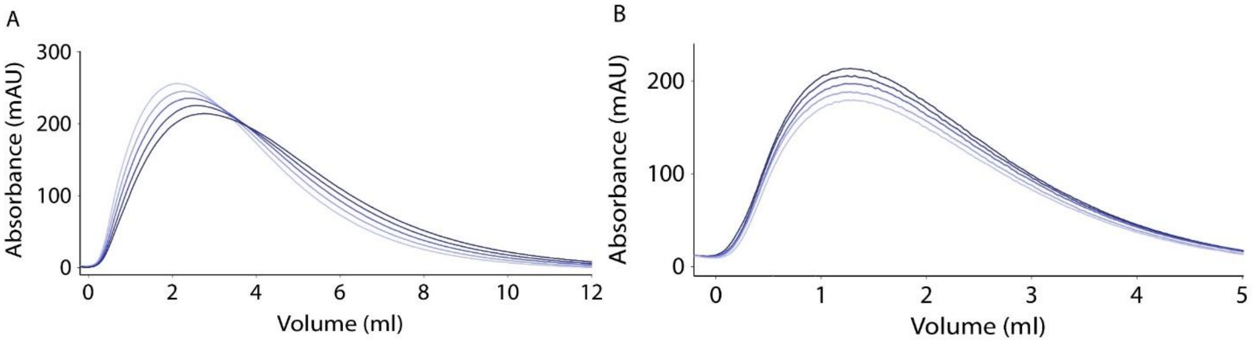

Figure S6. Z_{Ca} resin stability under long-term exposure to CHO cell supernatant represented by the amount of antibody that could be captured and eluted over time. A) Overlay of five consecutive purification cycles after ca 6 days, with increasing color brightness indicating increasing time. The elution profile changed with each cycle but did not affect the elution peak area. B) Overlay of five consecutive purification cycles after ca 8 days, using new supernatant with a different mAb concentration as compared to (A), with increasing color brightness indicating increasing time. A considerable decrease in peak area with each cycle implies a continuous decline in resin capacity.

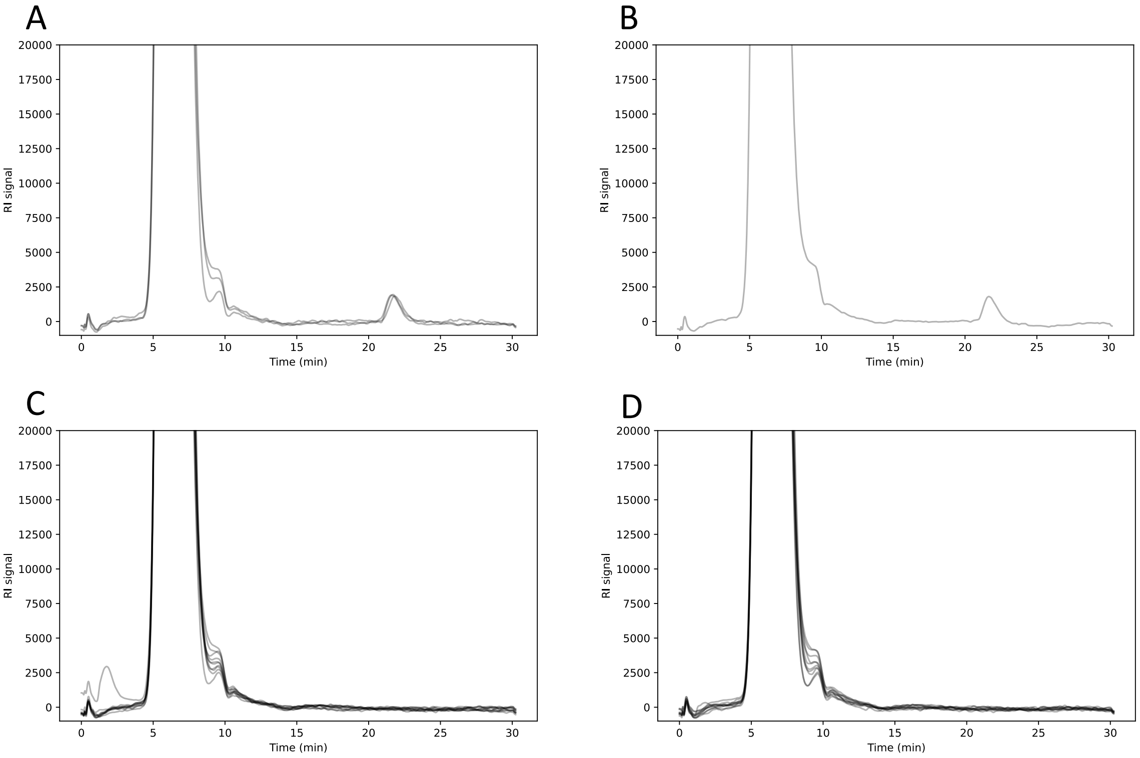

Figure S7. Overlapped chromatograms from the RP-HPLC analyses for the detection of Tnbp corresponding to different days of the pilot-scale run. A) Product from the VI step, B) Flowthrough from the CEX column in the loading phase, C) Product from the CEX step, D) Product from the AEX step. Tnbp elutes at a retention time between 21 and 23 min.

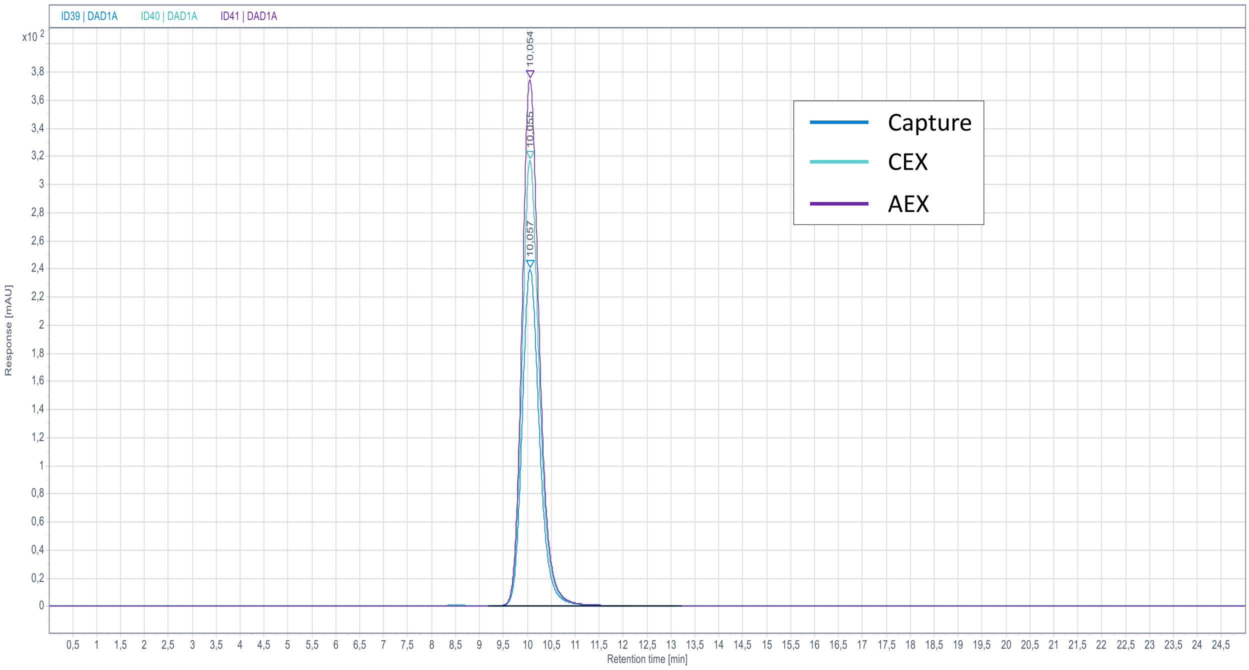

Figure S8. Chromatograms from the SEC experiments of the capture (blue), CEX (turquoise) and AEX (violet) elution pools at a wavelength of 280 nm for the analysis of mAb aggregates, corresponding to cultivation day 14 of the pilot-scale run.

Supplementary Tables

Table S1. Column volumes for the chromatography steps.

Step	Protein load (mg/mL)	Small scale				Pilot scale			
		Mass load[a] (mg)	Resin volume (mL)	Column volume[b] (mL)	Column dimensions[c] (cm x cm)	Mass load[a] (mg)	Resin volume (mL)	Column volume[b] (mL)	Column dimensions[c] (cm x cm)
Capture	31.7	8	0.25	1	0.7 x 2.5	1188	37	57	2.6 x 10.8
CEX	44.8	24	0.53	2	0.7 x 5.0	3563	80	120	5.0 x 6.1
AEX	89.6	24	0.27	1	0.7 x 2.5	3563	40	60	2.6 x 11.3

[a] Nominal harvest concentration of 1 mg/mL. Harvest flow rate of 1.425 vvd (corresponding to a perfusion rate of 1.5 vvd with 5% bleeding). Cycle time of 40 min for capture and 120 min for the polishing steps.
[b] Considering a column void of 34%. Minimum column volume was 1 mL.
[c] HiScale™ 26/40 and 50/20 columns were used at pilot scale. HiTrap™ 1 mL columns were used at small scale.

Table S2. Overview of the perfusion strategy in the seed bioreactor (N-1) and production bioreactor (N)

Bioreactor	Day	VCD [10^6 cells/mL]	Perfusion rate [vvd]	Average CSPR [pL/(cell*day)]	$Glucose_{in}$* [mM]	Medium	q_glc [pmol/(cell*day)]
N-1	0 - 2	1.5 - 5	0	0		HA	3.9 → 1.9
N-1	2 - 3	5 - 9	0.25	33	33	HA	1.1
N-1	3 - 7	9 - 70	0.45 - 1.6	33	33	HA	1.1
N	0 - 1	10 - 15	0.3	25	45	HA-7a/3-7b/1	1.9 → 1.1
N	1 - 4	15 - 70	0.5 - 1.5	25	45	HA-7a/3-7b/1	1.1
N	4 - 5	70 - 100	1.5	18	73	HA-7a/6-7b/1	1.2
N	5 - end	~100	1.5	15	73	HA-7a/6-7b/1	1.0 - 1.1

*Concentration in the fed medium including feeds/additives
